# Toward more economical large-scale foundation models: No longer a game for the few

**DOI:** 10.1016/j.xinn.2025.100832

**Published:** 2025-01-30

**Authors:** Yiqing Wu, Zhao Zhang, Fei Wang, Yongjun Xu, Jincai Huang

**Affiliations:** 1 Institute of Computing Technology, Chinese Academy of Sciences, Beijing 100190, China; 2 College of Systems Engineering, National University of Defense Technology, Changsha 410073, China; 3 Lab of Big Data and Decision, National University of Defense Technology, Changsha 410073, China

## Introduction

**Figure 1:**
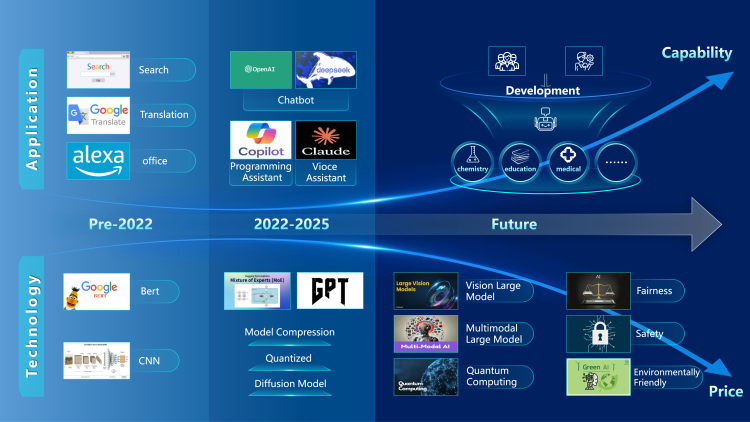
Toward more economical large-scale foundation models

In recent years, the development of large-scale foundation models (LFMs) has made great advances. However, the high training costs and computational demands have long been a bottleneck for the widespread adoption of this technology. With technological advancements, this situation is undergoing a fundamental transformation. The recent release of DeepSeek-V3^1^ has sparked extensive discussions. Through innovative architectural design and efficient training strategies, it has significantly reduced training costs while achieving performance comparable to top-tier closed-source models. The pre-training cost of DeepSeek-V3 is only $5.576 million, far lower than the hundreds of millions of dollars required for models like GPT-4. As shwon in Figure 1, this breakthrough not only marks the democratization of LFM technology but also opens up opportunities for more small- and medium-sized enterprises and research institutions to participate in AI innovation. In the future, LFMs will no longer be a game for the few.

## Large-scale models are becoming smarter but cheaper

The development of large-scale model technology is currently at an exciting time: on the one hand, its performance has achieved significant breakthroughs across multiple domains, and on the other hand, the significant reduction in usage costs is reshaping its application. This synergistic effect of performance enhancement and cost reduction is opening up new possibilities for the widespread adoption and application of AI technologies.

From the perspective of technological advancements, large-scale models have demonstrated remarkable progress across various fields. In terms of mathematical abilities, the accuracy in the GSM8K mathematical problem-solving test skyrocketed from 50.9% for Llama-1 to 96.8% for Llama-3.1.^2^ This indicates that AI now possesses a substantially solid capability for mathematical reasoning. In the programming domain, the HumanEval test score improved from 23.7% to 89%,^2^ signifying that AI not only understands code but can also write high-quality programs. The progress in knowledge comprehension is equally significant, with the MMLU test score rising from 63.4% to 88.6%, suggesting that large models are becoming increasingly adept at acquiring and applying knowledge. All these signs indicate that large models are evolving from mere “imitators” into genuine “thinkers.”

Meanwhile, the cost of using large-scale models is rapidly declining. For instance, ZhiPu AI recently reduced the price of GLM-3-Turbo from $0.7 per million tokens to $0.14, a staggering 80% reduction; the price for batch processing is even lower at $0.007 per million, meaning that just $1 can process 14 million tokens. Additionally, DeepSeek has slashed its application programming interface (API) input price from $0.14 to $0.014 per million tokens, a 90% reduction, making it only 1% of the cost of OpenAI’s GPT-4o. This significant drop in cost not only lowers the technical barriers but also provides more opportunities for small- and medium-sized enterprises and individual developers to utilize large models.

## Behind the phenomenon is technological advancements

The key point for the reduction in the cost of large-scale models is not market competition but rather technological advancements. The compute overhead associated with LFMs primarily involves two aspects: the first is training, which involves creating an LFM. The next is inference, which pertains to using an LFM. Through innovations in model architecture, advancements in model compression techniques, and upgrades in computational cost efficiency, the compute cost is significantly reduced in both the training and inference stages. Thus, the price of LFMs rapidly decrease.

(1) Advances of model architecture: the recent release of DeepSeek-V3 has garnered widespread attention due to its remarkably low training costs, revealing significant advancements in LFM technology. DeepSeek-V3 is the first to adopt FP8 mixed precision for training, theoretically reducing computational resource consumption (both from memory and computation time) to just 50% of that of mainstream FP16 models. Additionally, in terms of model architecture, DeepSeek-V3 incorporates mixture of experts (MoE) and multi-head latent attention (MLA)^3^ technologies. With MoE,^4^ DeepSeek-V3 activates only 5% of its parameters during each computation, which means the computation speed has increased by 20 times. The MLA technology also significantly reduces the computational overhead of the key-value (KV) cache, a critical component in LFM inference. These innovative approaches not only enhance the model’s output efficiency but also drastically lower training costs. As a result, the API price of DeepSeek-V3 is only 1% that of GPT-4o, a model with comparable performance. In the future, innovations in neuromorphic chips, quantum computers, and superconducting computing structures are expected to further enhance computational power and reduce the cost of LFMs.
(2) Advances in model compression techniques: through techniques such as pruning, quantization, and knowledge distillation, it is possible to significantly reduce the number of model parameters and computational complexity without substantially compromising performance. For instance, SparseGPT technology can increase the sparsity of GPT-series models to 50%–60% in a single pruning step without the need for retraining, thereby significantly lowering computational costs. Additionally, quantization techniques further reduce storage and computational demands by converting high-precision parameters into low-bit representations. The application of these technologies enables large models to run efficiently on resource-constrained devices, thereby reducing deployment costs.
(3) Improvement of computational cost efficiency: in recent years, the release of GPU series such as A100, H100, and H200 has significantly improved the computational efficiency, increasing from 19.5 to 67 TFLOPS. Additionally, H100 and H200 support FP8 data formats and transformer engines, further optimizing the efficiency of model training and inference. The growth in computational power has drastically reduced the time required to train large-scale AI models. For example, training LLaMA1 required 1 million GPU h per trillion tokens, while LLaMA3 only requires 420,000 GPU h per trillion tokens.

## Now, future, and challenges

### Now

With the improvement in large language model capabilities, many practical applications of large language models have now emerged.^5^ These models have been widely deployed across various fields, including healthcare, urban computing, retail, and scientific research. For instance, Amazon’s programming assistant, Copilot, now boasts over 1.3 million paid users and 50,000 enterprise users. Social investigation shows that 70% of large language model users in China utilize large language models at least once in an average workday. However, the majority of LFM services are currently dominated by tech giants, resulting in limited innovation and application opportunities for small- and medium-sized enterprises.

### Future

As affordable APIs for LFMs become more widespread, the challenges of computational power and training costs will cease to be obstacles. In the future, LFMs will no longer be a game for the few. Instead, they will emerge as a vital technological force accessible to the general public. By utilizing inexpensive LFM APIs to develop intelligent agents, small businesses and even individuals will be able to easily create personalized AI tools for various applications. This will enable LFMs to profoundly impact people’s lives, much like the Internet has done. What is even more promising is that this technological democratization will accelerate the deep integration of AI for scientific research (AI4Science) and reshape the paradigm of knowledge innovation. In the future, AI agents may further break through the traditional boundaries of scientific research and play a more significant role in fields such as biomedical research, chemical materials, and archaeological discoveries. Besides, current innovations mainly focus on large language models; future multi-modal and vision models may leverage these innovations to cut costs.

### Challenges

However, LFMs currently still face multiple challenges. First is the depletion of training data. Existing models have nearly exhausted all publicly available high-quality data, making it increasingly difficult to acquire enough data for further large-scaling model. The next step might involve the use of artificially synthesized data; however, how to synthesize high-quality training data remains a challenge. Second is the computational resource barrier. While computational demands have significantly decreased, access to advanced computing power remains critical. A few countries or technology giants may monopolize global high-end computing resources through their financial and technological advantages, creating computational barriers. This monopoly could stifle innovation in smaller enterprises and research institutions while hindering broader model deployment. Third is the stagnation of the foundational model’s architecture. Despite recent incremental improvements, most research still focuses on optimizing transformer architectures rather than pursuing revolutionary breakthroughs. Transformers face inherent limitations in processing long sequences, integrating multi-modal data, and enabling efficient inference alongside issues like high computational complexity and energy consumption. Besides, with models becoming widely adopted, issues such as regulatory compliance, alignment with human values, security risks, and intellectual property protection require urgent resolution. Finally, the energy consumption of large models is significantly higher than that of traditional AI models, and the resulting energy consumption and ecological issues are also points of concern.

## Conclusion

The rapid development of LFMs signifies the entry of AI into a new era. LFMs are now demonstrating a trend of continuously improving performance and decreasing costs. Innovative technologies like DeepSeek-V3 are driving the democratization of LFMs, benefiting more small- and medium-sized enterprises and developers. However, challenges such as data exhaustion, computational barriers, and architectural innovation still need to be addressed. Additionally, ethical and social implications, such as data privacy and algorithmic bias, cannot be overlooked. In the future, through technological innovation and collaboration, we anticipate achieving a more inclusive and sustainable AI development that advances technology for the benefit of society as a whole.

## Acknowledgments

This work is supported by the 10.13039/501100001809National Natural Science Foundation of China under grant nos. 62206266 and 62372430 and the 10.13039/501100004739Youth Innovation Promotion Association CAS no. 2023112. The funders had no role in study design, data collection and analysis, decision to publish, or preparation of the manuscript.

## Declaration of interests

Yongjun Xu is an Editorial Board member of The Innovation and was blinded from reviewing or making final decisions on the manuscript. Peer review was handled independently of this member and their research group. The other authors declare no conflicts of interest.
